# Insight into Natural History of Congenital Vitiligo: A Case Report of a 23-Year-Old with Stable Congenital Vitiligo

**DOI:** 10.1155/2017/5172140

**Published:** 2017-08-14

**Authors:** Chelsea Casey, Stephen E. Weis

**Affiliations:** Department of Internal Medicine, University of North Texas Health Science Center, Texas College of Osteopathic Medicine, Fort Worth, TX, USA

## Abstract

Vitiligo is a disorder of skin pigmentation. It affects approximately 1% of the world's population. Vitiligo occurs equally between the sexes with no racial predilections. The majority of cases are acquired and arise between the second and third decades of life. Acquired vitiligo has an unpredictable clinical course. Congenital vitiligo is rare with few reported cases. Due to the rarity of congenital vitiligo, little is known about the clinical course. For patients with acquired or congenital vitiligo, the psychosocial burden can have a profound impact on quality of life. The unknown course of congenital vitiligo can exacerbate the feelings of distress and embarrassment. We report of a case of congenital vitiligo that has been stable for 23 years. The patient had no associated autoimmune disease. The pathogenesis of congenital vitiligo is unknown. This case may be useful to assist clinicians caring for newborns with congenital vitiligo in reassuring parents.

## 1. Introduction

Vitiligo is a benign disorder of skin pigmentation with a clinical presentation of white macules or patches [1]. It affects approximately 1% of the world's population with most cases appearing in the second and third decades of life. Congenital vitiligo is rare with few reported cases. The etiology of vitiligo is unknown. It is believed to be multifactorial with hypotheses regarding genetics, environment, neurogenic, and autoimmune components. Acquired vitiligo has an unpredictable clinical course with subtypes that include nonsegmental, segmental, or mixed vitiligo. Due to the rarity of congenital vitiligo, little is known about its clinical course. In our research, only 6 cases of congenital vitiligo are reported. However, not all case reports comment on the clinical course of the lesions. Although benign, the psychosocial burden can have a profound impact on quality of life for patients with acquired or congenital vitiligo. The lack of knowledge on the clinical course of congenital vitiligo can intensify the feelings of distress. We present a case of a 23-year-old female patient who was found to have three stable vitiligo patches since birth without any spread of existing lesions or occurrence of new lesions.

## 2. Case 

A 23-year-old Caucasian female presented to the dermatology clinic for multiple dermatologic issues. One of her findings was three depigmented patches. According to her history, and confirmed by her mother, she was born with the three skin lesions. The three lesions had never altered in size or pigmentation, were not painful, and had never blistered. She and her mother denied disease progression since birth. Her prior medical history was only significant for eczema and contact dermatitis. There was no personal or family history of autoimmune diseases, skin malignancies, or trauma.

**Figure 1:**
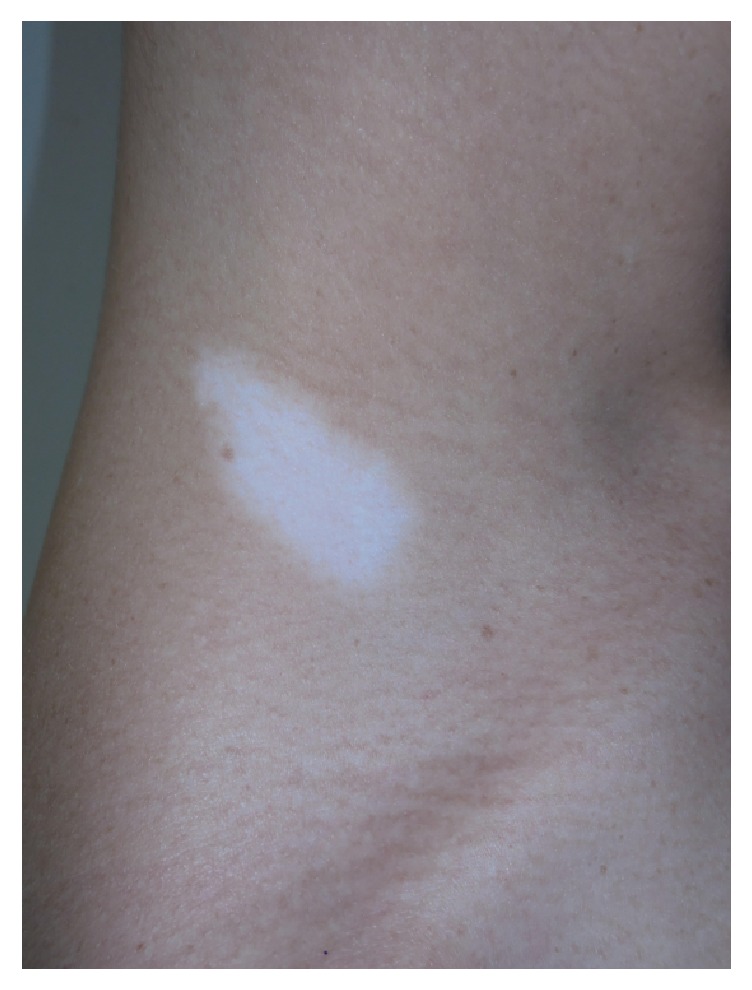
Depigmented patch with leukotrichia right inferolateral neck.

**Figure 2:**
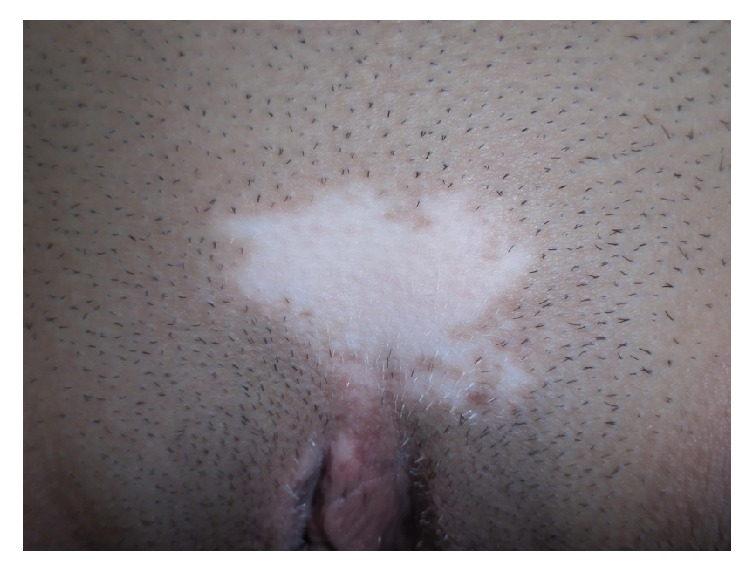
Depigmented patch with leukotrichia on mons pubis.

**Figure 3:**
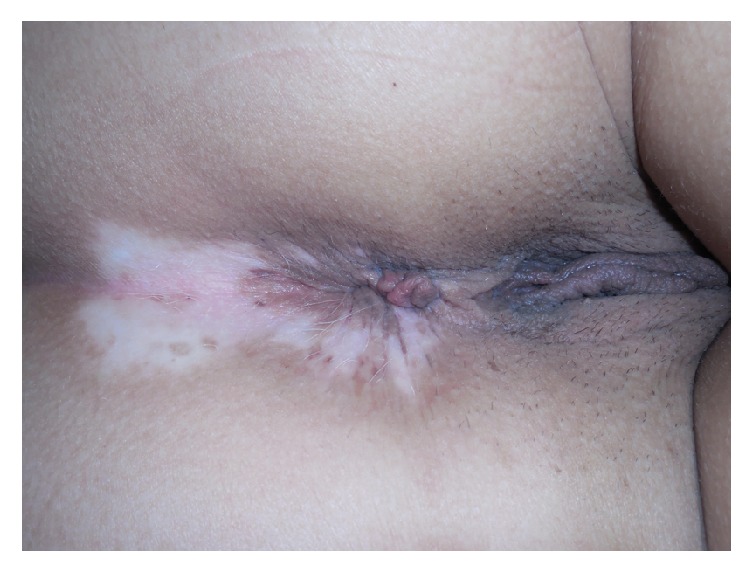
Perianal depigmented patch with leukotrichia.

On physical exam, she had an approximately 3 cm depigmented patch with sharply defined borders on her right inferolateral neck with leukotrichia (Figure 1), a 4 cm depigmented patch with sharply defined borders and leukotrichia on the mons pubis (Figure 2), and a perianal 6 cm depigmented patch with sharply defined borders and leukotrichia (Figure 3). Considering the clinical history and physical exam findings, the diagnosis of stable congenital vitiligo was given. During the encounter, the patient expressed significant distress, even to the point of being tearful. She was concerned about the inability to obtain a life partner due to her perceived disfigurement. Etiology, clinical course, and treatment were discussed. The patient elected to not seek treatment at the time of presentation as the original three lesions had remained static since birth and she had no new lesions.

## 3. Discussion

Skin pigmentation occurs by melanocytes that are within the epidermal basal layer. Vitiligo is a skin pigmentation disorder in which the melanocytes are affected. It is traditional in histopathology that vitiligo is characterized by an absence of functioning melanocytes. On developing lesions, an infiltrate of lymphocytes is frequently identified [2]. Clinically, it is manifested by depigmented macules and patches [3].

Although vitiligo has been known for a significant portion of human history, its etiology has remained obscure [4]. Multiple theories have been proposed, which include genetic, environmental, and autoimmune mechanisms. Vitiligo has a 7- to 10-fold increased risk in first-degree relatives. There is high occurrence of comorbid autoimmune diseases such as Hashimoto's disease and Diabetes Mellitus in patients with vitiligo [1]. In 1995, a case of a 31-year-old male with congenital vitiligo who subsequently developed multiple sclerosis is reported [5]. Studies on vitiligo genetics have shown 36 convincing nonsegmental susceptibility loci [4]. Approximately, 10% of genes within or near these loci encode melanocyte proteins. It is theorized that melanocyte proteins might act as autoantigens [6]. Development of vitiligo is believed to be interplay between several factors, including autoantigens and a trigger. Certain triggers, such as oxidative stress or physical trauma, expose antigens and lead to an autoimmune response [7]. It is also postulated that the immune attack may begin in utero in genetically susceptible individuals [6]. There is a single case report of a male child born with congenital vitiligo and his gestation was complicated by his mother acquiring new onset vitiligo [8]. Although a lot has been gleamed from recent advances in vitiligo pathophysiology, the influence of various factors is still poorly understood.

In an attempt to cohesively define nomenclature, clinical progression, outcome, and disease classification, a review was conducted by* Vitiligo Global Issues Consensus Conference [9].* Vitiligo can be classified as nonsegmental, segmental, or mixed [7]. The subtypes are important for clinical symptoms and etiology [4, 9]. Nonetheless, vitiligo has an unpredictable clinical course. Some lesions remain stable, while others slowly progress, new lesions may appear, or some patients experience flares in between stable periods [10, 11].

Vitiligo can have a significant impact on quality of life due to its psychological aspects [3]. As our patient expressed, individuals often feel a significant burden with low self-esteem due to stigmatization. Our patient with three small, stable lesions that are normally covered with clothing had strong feelings that this would affect her ability to have a normal life. Women and children are often the most impacted by the feelings of embarrassment [12]. Since congenital vitiligo is a rare diagnosis, the inability to provide information on the clinical course further exacerbates the stress of the diagnosis for the patients and their families. Therefore, patients with congenital vitiligo have similar quality of life issues to those with acquired vitiligo.

Of the 6 previously reported cases of congenital vitiligo [6, 11, 13], only one reported the clinical course of vitiligo. The 71-year-old man whose gestation was complicated by his mother acquiring vitiligo had very minimal changes over the course of his life [8]. We report a case of congenital vitiligo that has been stable for 23 years. The patient had no associated autoimmune disease. The pathogenesis of congenital vitiligo is unknown. This case may be useful to assist clinicians caring for newborns with congenital vitiligo in reassuring parents.
